# On Toothache

**Published:** 1876-07

**Authors:** 


### SELECTED ARTICLES.

---

### ARTICLE IV.

### *On Toothache.*

Dr. Duckworth recently recommended, in the *Practitioner*, the application of a solution of bicarbonate of soda to relieve the pain of toothache. Suggested by this, Mr. J. Smith Turner, dental surgeon to the Middlesex Hospital, writes an article of considerable interest and utility.

He holds that while the employment of bicarbonate of soda will not unfrequently be attended with great benefit, to suppose its application will invariably produce the desired results may lead to unreasonable disappointment. The term toothache is applied indiscriminately to all pain situated in or around the teeth, but the disturbance may arise from different causes. The pain in the case alluded to by Dr. Duckworth evidently arose from the covering of the tooth-pulp being insufficient to protect it from the action of the saliva, or from the exposure of the dentine to the secretion of the mouth through the loss of its natural covering, the enamel. Hence the subsidence of pain on the use of the antacid. The same application is of great use where the enamel structure is feeble, and where numerous defective spots are present, as is frequently seen in young phthisical patients; also in children where there is a general defective condition of the first teeth, proceeding, it may be from neglect or from defective development, or from some disease of the mucous membrane; and in pregnant women, in whom the teeth are frequently found decaying round the base of the crowns in a line with the margin of the gum. That the toothache from which such subjects suffer is due to a vitiated condition of the fluids of the mouth may be inferred from the sudden access of pain so frequent after eating or during sleep, and which is so often ascribed to increase of temperature, or to the increase of circulation in these parts owing to the recumbent position, but which is speedily relieved by the use of a tepid solution of soda bicarbonate. Mr. Turner then continues: Some of the conditions inducing toothache are equally patent or equally obscure to the general practitioner and to the specialist. Ulceration of the membranes of the mouth, for example, would be at once observed, while irritation of the dental nerve, in the absence of a visible cause, could only be diagnosed after careful and extended observation, and perhaps some unsuccessful efforts in treatment. There are, however, conditions, and suffering and consequent constitutional disturbance, which the general practitioner should be able to ameliorate until such time as special skill be available. A decayed tooth may give pain although the tooth-pulp be not exposed. The alkaline lotion will not give relief, and if the saliva be tested it may be found normal. The cause of the pain must therefore be sought in the tooth itself. The decayed dentine is an irritant; this ought to be removed at least partially if not entirely. To do this without exposing or wounding the tooth-pulp is a delicate operation, and a man not in daily practice could not be expected to accomplish it completely; still enough may be done to serve the immediate purpose. A small mouth-glass and a few excavators, such as are to be had at any dental depot, are all that are required in the way of instruments. Their cutting edges should be round or spoon shaped—if they have any sharp angles they are much more likely to wound the tooth-pulp. The cavity should be syringed with tepid water, and that may be sufficient; but there is generally a quantity of soft dentine which should be removed if possible. The cavity should be dried out with cotton, wool or some other absorbent, and a small pellet of wool moistened with carbolic acid and glycerine, should be placed in it, and over this a piece of wool partially moistened with mastic (white and hard varnish answers admirably) should be packed. The packing may be accomplished with a blunt probe, and the pressure should be light aud not in the direction of the pulp cavity. This will serve till a permanent plug can be introduced, but should not be trusted beyond two or three days, especially in cavities between the teeth.

If the cavity be on the masticating surface of a tooth the wool should be free from pressure on the occlusion of the antagonising teeth. If it be an interstitial cavity, the gum beyond the margin of the cavity should be disturbed as little as possible, unless it has growu into it, when the wool should be packed with a view of pushing the gum out. If the margin of the gum be left projecting into the cavity its secretion will become abnormal, owing to the irritation caused by the wool; the cavity will be inundated with the secreted fluid, which will have no way of escape, and the discomfort of the patient thereby aggravated rather than relieved. If possible the wool should not be allowed to depend upon support from the adjacent tooth for retaining its position, as the pressure is likely to separate the teeth, when the plug will leave the walls of the cavity, and so matters will return to their original condition. The wool used for this purpose should be deprived of its greasy character; hence the pink wool, which has been cleansed before dyeing, is best for use.

Toothache may arise from an exposed tooth-pulp, and in such a case the same course of syringing and cleansing should be pursued as already laid down, and some application used which will subdue the irritation of the pulp, applied as in the former instance, and covered over with wool and mastic. Creasote is an old and deservedly a favorite remedy for such a condition of things, but it should be pure wood creosote, as that which is made from coal-tar is very likely to act as an irritant. The following mixtures are recommended for use in place of creasote, and if complication be a merit they have that advantage:—

℞ Acidi carbolici solutionis saturatæ

Chloral hydratis sol. sat.

Tinct. camph. co.

Ext. aconit. fluid, aa 1 ounce.

Ol. menth. pp. ½ ounce.

℞ Chloral hydrat., 1 drachm.

Aqua fl. ½ ounce. Misce et adde.

Tinct. aconiti (Fleming,) 15 drops.

Chloroformi.

Ætheris.

Spt. vin. rect., aa 20 drops.

R Liq. opii. sedativ,

Ol. caryophyll. aa 3 ounces.

Camphor, 1½ drachm.

This last I have found very useful.

Pain may arise from the inflammation of the periosteum, and may be situated in an otherwise healthy tooth which has been jarred or wrenched ; such cases are not uncommon in the game season from shot or bone splinters getting between the teeth during mastication. Or it may come from a tooth carrying a large mass of metal stopping having been subjected to unusual conditions, such as exposure of the side of the face next which it may be situated in riding against wind or rain. A low state of health, constipation, exhaustion after violent exercise or prolonged occupation, rheumatism, scrofula, or syphilis may all produce inflammation. The gum surrounding the affected tooth is tender to the touch, and becomes elongated and loose. The degrees of inflammation are various, and in its early stages may be cut short by wiping the gum dry, and frequently applying tincture of iodine of double strength all over the inflamed part. A piece of cotton wool soaked in water as hot as can be borne, and laid between the gum and the cheek, makes an excellent poultice, and if accompanied by a slight aperient, is almost sure to give relief in a chronic case. The constitutional treatment required must be obvious to medical men, who have much more command over their patients in the administration of general remedies than the dentist; but I may mention that that there is no medicine more likely to cut short in its early stage an acute case of periostitis connected with the teeth than five grains of pil. saponis co. Two leeches applied to the gum over the affected tooth have repute for doing good, but in some cases prove very disappointing. If there be marked swelling of the gum towards the apex of the affected tooth, lancing is the best thing that can be done, but to be effectual it must be done thoroughly. The instrument should be strong as well as sharp, and capable of cutting through the alveolar plate between the gum and the tooth. Before lancing, Mr. Tomes recommends that the gum should be painted with equal parts of tincture of iodine of double strength and Fleming's tincture of aconite. Teeth may become tender around the neck from recession of the gums, or from an artificial case of teeth being attached to them. The exposed parts of the tooth should be cauterized with nitrate of silver, and if a metal plate have to be worn again immediately a layer of tissue paper ought to be placed between the cauterized surface and the metal. As the nitrate of silver should be allowed to remain on the tooth a few minutes in order to prove effectual, the cheek and tongue and saliva should be kept away from it as much as possible by holding some ordinary cotton wool round the tooth. When the wool is withdrawn a strong solution of salt should be used immediately, to convert any free nitrate into an inert chloride. Unfortunately the nitrate of silver cannot well be used on the necks of front teeth, where a ring of sensitive decay is often found, but it is a valuable remedy where appearance is not in question.

The after pain of an extraction may be modified by washing away the blood-clot and lightly plugging the alveolar cavity with wool saturated with

℞ Acidi carbolic, glacialis,
Liq. potassæ, aa 1 drachm,
Aquæ dest., 1 ounce, as recommended by Mr. Tomes in his "System of Dental Surgery."

From the foregoing remarks it may be inferred that there are degrees of inflammation of the tooth-pulp and of the periosteum. As the treatment of subacute inflammation of the tooth-pulp is very limited and quite incomplete unless the tooth be properly plugged, so in cases of acute inflammation of that organ, the general practitioner can only relieve the patient temporarily, that is, if the tooth is to be saved. It may be well, however, to point out that subacute inflammation may arise from injury by mechanical violence or from the masticating surface of a tooth being denuded of enamel even to a very small extent. The tooth becomes troublesome, and frequently reminds its owner of its existence when subjected to thermal changes,or even the ordinary work of mastication. If, on careful examination of a tooth so affected, there be no signs of structural defect observed, search should be made for a decayed tooth elsewhere. When this is found, a process of examination, such as tapping with an instrument, or probing the decayed part, or directing a stream of cold water into it, may start all the symptoms complained of in an intensified form. In rheumatic people and people under the influence of mercury, the irritable state of the teeth is often found. In acute inflammation of the tooth-pulp the history generally extends over a long period. Different substances have annoyed a tooth in which a cavity has been known to exist a long time but which, according to the patient, has always remained the same. Sweets or bitters, heat or cold have every now and then caused uneasiness, but when these disturbing causes have been removed the pain has ceased. But at length the periods of cessation have diminished, and the length and intensity of the attacks have increased, the pain radiates from the tooth to the other teeth and over the side of the face, and assumes a throbbing character. These attacks last several hours and then suddenly subside, but surely to return again, sometimes without the smallest apparent provocation, or if the patient lie down. This will go on for a shorter or longer period and with varying intensity, according to the constitutional state of the patient till the pulp dies. The next state of the tooth is the commencement of an alveolar abscess, which, if not attended to may involve the removal of the tooth and even of a portion of the alveolar plate, or even further mischief.

In chronic inflammation of the tooth-pulp the pain is less regular in its advent, shorter in duration, and less severe than in acute cases. The peculiarity of most importance is the straggling neuralgic pain which is rarely referred to a definite centre. If any tooth be specified as its seat, it is not unlikely to be a sound one, but even its being decayed is not sufficient in itself to condemn it. In fact, the tooth which is nearly destroyed by caries is not so likely to be the offender as one which is in a better state of preservation. The careful application of a blunt probe to the floor of the cavity will readily detect the irritated nerve, which should be treated as already described, or the tooth removed if worthless.—*London Chemist and Druggist.*

---

